# Homecare protective and risk factors for early childhood caries in Japan

**DOI:** 10.1186/s12199-018-0746-8

**Published:** 2018-11-06

**Authors:** Ritsuko Nishide, Mayumi Mizutani, Susumu Tanimura, Noriko Kudo, Takayuki Nishii, Hiroyo Hatashita

**Affiliations:** 1 Course of Nursing Science, Mie University Graduate School of Medicine, 2-174 Edobashi, Tsu, Mie 514-8507 Japan; 2 Department of Nursing, Faculty of Nursing, Kansai University of Health Sciences, 2-11-1 Wakaba, Kumatori, Sennan, Osaka, 590-0482 Japan

**Keywords:** Early childhood caries, Tooth brushing, Bottle feeding, Dental plaque, Mother’s caries, Longitudinal studies

## Abstract

**Background:**

Early childhood caries (ECC) affects children across Japan and throughout the world. Thus, it is important to identify dietary and dental care habits that either promote oral health or cause ECC. The objective of this study was to identify protective and risk factors associated with ECC in Japan.

**Methods:**

In a typical rural Japanese community, we selected children born between 2004 and 2008 who had received checkups at their community health center including oral examinations conducted by dentists. We obtained data from children’s records and from a questionnaire filled out by parents. We enrolled only children who at their checkup for 18-month-olds had no caries, and we obtained data about them at their checkup for 3-year-olds. We classified children as either having caries (treated or untreated) or being caries-free. We conducted bivariate analyses using data on child/family demographic characteristics, child’s dietary habits, and child/parental oral health habits. We also conducted logistic regression analysis to control for variables and identify predictors of the presence/absence of caries.

**Results:**

Five hundred sixty six children (278 boys, 288 girls) were enrolled and followed. After 2 years, 173 children (30.6%) presented with caries. Logistic regression analysis predicting caries at follow-up identified the interaction term “bottlefed overnight and brushed irregularly” at 18 months of age as a highly significant predictor of developing caries—adjusted odds ratio (AOR) of 14.27, 95% confidence interval (CI) 1.02–199.71. Two variables measured at follow-up were also significant predictors: having low levels of dental plaque (AOR 2.41, 95% CI 1.34–4.35) and having a mother who had untreated caries (AOR 1.84, 95% CI 1.09–3.12).

**Conclusion:**

Public health efforts should encourage parents to eliminate bottle feeding overnight and promote brushing twice daily as children’s teeth begin to erupt. Greater efforts should be made to teach parents and daytime caregivers how to brush effectively to remove all plaque. Health professionals should pay close attention to mothers’ oral health status. Mothers with caries should receive prompt treatment and be assisted in developing better dietary and oral health habits that will benefit themselves and their children. Policies and programs should focus more on family oral health rather than just child oral health.

## Background

Early childhood caries (ECC) remains a significant public health problem in Japan. The most recent survey of ECC in Japan conducted in 2014 estimated the prevalence of caries among 3-year-olds at 17.69% [1]. While the overall prevalence rate of ECC among 3-year-olds in Japan has declined steadily from 55.8% in 1989 and is lower than rates in Canada, Taiwan, and Australia [2], within Japan there are substantial regional disparities in the prevalence of ECC [1, 3]. A study of ECC in Japanese cities and prefectures found that among 3-year-olds, prevalence rates ranged from 6.08% to 31.15% [1].

The American Academy of Pediatric Dentistry defines ECC as “the presence of 1 or more decayed (non-cavitated or cavitated lesions), missing (due to caries), or filled tooth surfaces in any primary tooth in a child under the age of six” [4]. In a recent longitudinal study, ECC was found to be strongly associated with caries in permanent teeth [5]. Primary teeth with untreated caries have been shown to be associated with delayed development of permanent teeth [6, 7]. The Japan Dental Association classifies caries into 5 types for 18-month-olds (O_1_, O_2_, A, B, C) and 5 types for 3-year-olds (O, A, B, C_1_, C_2_) [8].

The enamel of primary teeth is thin and susceptible to damage from contact with acidic foods and drinks [9]. Individual-level risk factors for developing ECC include children consuming sugary snacks and/or beverages regularly [10–12], primary caregivers not initiating brushing before a child’s first birthday [13] and primary caregivers not brushing 18-month-old children’s teeth daily [10]. Some studies have also shown that the time and duration of breastfeeding can be a risk factor for ECC among children who began to eat solid foods [14–17]. Sleeping with a bottle has also been identified as a risk factor for ECC [16, 18].

ECC can be prevented through healthy eating habits, brushing twice daily and flossing daily, receiving regular preventive dental services, and community-level behavioral education [13, 19, 20]. Studies on the prevention of ECC have shown that community-level education about dietary habits and dental care is as important as measures taken every day at home (i.e., brushing and flossing) [21, 22]. Several studies have shown that the etiology of ECC is often multifactorial and associated with multiple risk factors [23]. Few studies have examined the interactions between individual factors as predictors of ECC in Japan or elsewhere. ECC occurs at an important stage of development when children are establishing their dietary and oral health habits. Young children tend to eat frequently throughout the day, thus putting their teeth in contact with substances that can promote the development of plaque. Bottle feeding and breastfeeding are important factors, but they are not the only factors [23]. The interactions of improper dietary habits and poor dental care habits are important to consider [24]. Establishing and maintaining good dietary and oral health habits while minimizing bad habits has been shown to reduce the occurrence of ECC [23].

Throughout Japan, community health centers offer low-cost health checkups for 18-month-olds and 3-year-olds that include oral health examinations. In 2016, nationwide community health centers conducted checkups on 96.4% of 18-month-olds and 95.1% of 3-year-olds [25]. Still, in the Japanese context, there are significant questions about the contributions and interactions of child-related factors and maternal-related factors to ECC. No longitudinal studies have examined the combination of dietary and oral health habits among young children in Japan. Furthermore, only a few population-based studies of children who are the same age have been published [10, 18]. These studies treated dietary habits and oral health habits as separate variables and did not examine interactions. To provide parents and daytime caregivers with useful information about how to care for young children’s teeth each day, it is important to have evidence about the effects of dietary and oral health habits over time. Therefore, we conducted a prospective longitudinal study to identify individual-level risk factors associated with the occurrence of ECC in Japan.

## Methods

### Study site and participants

We conducted our study in Mie Prefecture in a rural community that was similar to rural communities throughout Japan. The prevalence of ECC in 18-month-old children was equivalent to the national prevalence rate (2.5% in 2009, 1.25% in 2014) [1]. The community we selected had a population of about 20,000. Children under the age of 5 accounted for about 5% of the population while about 30% of the population were adults over the age of 65. The number of annual live births had declined to less than 200 per year [26]. Residents were engaged primarily in farming, fishing, light manufacturing, civil service, service industry work, and studying. As in 99% of communities in Japan, the water supply in this community was not fluoridated [27–29].

In Japan, health checkups are recommended for children aged 18 months (no later than their second birthday) and for 3 years (no later than their 4th birthday). Checkups were conducted in this community as in all municipalities according to the Maternal and Child Health Law [30]. In our study community, the local public health office mailed a reminder notice to the parents or guardians of every 18-month-old and 42-month-old. This procedure was followed in cities and towns throughout Japan.

Over 95% of children in the study community received regular checkups for 18-month-olds and 3-year-olds at the local community health center. For this study, we selected a population of children who were born between April 1, 2004 and March 31, 2008 who had received health checkups including oral examinations at the community health center. All children enrolled in the study had received a dental checkup for 18-month-olds and for 3-year-olds. We obtained data from children’s health checkup records and from a self-administered questionnaire for health checkups that is used at each community health center throughout Japan, including at the local community health center where this study was conducted, using almost all of the basic question items developed by the Japan Ministry of Health, Labour and Welfare.

For each child, dentists examined the oral cavity using a dental mirror and they recorded their findings, including diagnoses, in the child’s dental checkup form. Dentists noted the status of dentition (e.g., erupted teeth and malocclusion), the presence and location of dental plaque, the presence and location of treated and untreated caries, and teeth missing due to caries or other causes. At the checkups for 18-month-olds (our baseline) dentists assessed the presence and degree of plaque accumulation and presence of caries on all surfaces of the maxillary central and lateral incisors. At checkups for the 3-year-olds, they examined all teeth for plaque and caries.

For inclusion in this study, we enrolled only children who at the time of their checkup for 18-month-olds had no caries. We reviewed the records for these children again at their checkup for 3-year-olds and classified them as either having caries (having untreated or treated carious teeth) or being caries-free (having healthy teeth only and no missing teeth due to caries).

Before or during the checkups, parents were asked to fill out a questionnaire for 18-month-old checkups or for 3-year-old checkups used at community health centers throughout Japan. The questionnaire contained questions about the demographic characteristics of the child (e.g., age, gender, birth order), the family (e.g., person who was the child’s primary daytime caregiver), child’s nutritional habits (e.g., snacking, drinking sugary drinks, breastfeeding overnight, bottle feeding at nighttime), and parental/child dental health practices (e.g., frequency of teeth-brushing, regular dental checkups, fluoride applications). Mothers were also asked to respond to questions on the questionnaire about their own oral health. Those mothers who indicated that they had any treated and/or untreated teeth were classified for the purposes of this study as having “caries experience.” After the primary caregivers filled out the questionnaires, public health nurses interviewed them to double-check and clarify their responses.

### Data analysis

To analyze data from the dental exams and responses to the questionnaires, we first calculated descriptive statistics for all variables. Then, we conducted bivariate analyses using a chi-squared test by comparing participants based on their dental status (caries vs. caries-free) at their 3-year-olds checkup against their dichotomized demographic characteristics, nutritional habits, and preventative dental practices. We then conducted multivariate analyses by specifying logistic regression models to identify predictors of the presence/absence of caries among 3-year-olds while controlling for potential confounding variables. Initially, we specified models by forcing variables into a model based on the literature and calculated the adjusted odds ratio (AOR) for each variable. Based on these results, we subsequently specified models using interactions between selected variables to identify the potential predictive value of interaction terms. The goal of our modeling was to maximize the overall predictive value of the model based on the Nagelkerke *R*^2^ statistic while including conceptually important variables regardless of the significance of their contributions. Statistical analyses were performed using IBM SPSS Statistics 24.0. Significance levels were set at *p* < 0.05 for all tests.

### Ethical considerations

Participation in the study was voluntary. Parents were given the option to opt out during recruitment. The local community public health office provided us with anonymized data that were extracted from children’s electronic medical records. The study protocol was approved by local community authorities and the Ethics Committee of the Graduate School of Medicine, Mie University in 2014.

## Results

We contacted the parents of children who received checkups for 18-month-olds and none objected to providing the information we requested. During the study period, 581 children underwent checkups for 18-month-olds. Of those, 15 children (2.6%) presented with carious teeth and were thus excluded from our study. Children who were identified as having malocclusion were included in the study because at 18 months of age malocclusion cannot be said to hinder dental caries prevention behavior. All remaining children were diagnosed with healthy erupted teeth. This resulted in a total of 566 children (278 boys, 288 girls) enrolled at baseline (see Table 1).

**Table 1:** Characteristics of children enrolled at baseline in checkups for 18-month-olds

	All children		Enrolled Baseline		After 2 years Follow-up	
Age (months): mean (SD) range	18.5 (0.69) 18–23		18.5 (0.69) 18–23		42.8 (0.83) 41–48	
	*n*	%	*n*	%	*n*	%
Gender						
Male	281	48.4	278	49.1	–	–
Female	300	51.6	288	50.9	–	–
Position in birth order						
First	262	45.1	258	45.6	–	–
≥Second	319	54.9	308	54.4	–	–
Number of siblings						
0	235	40.4	232	41.0	114	20.1
≥ 1	346	59.6	334	59.0	452	79.9
Mother is a primary daytime caregiver						
Yes	394	67.8	388	68.6	137	24.2
No	187	32.2	178	31.4	427	75.4
No answer	0	0.0	0	0.0	2	0.4
Grandparent is a primary daytime caregiver						
Yes	231	39.8	227	40.1	441	77.9
No	350	60.2	339	59.9	123	21.7
No answer	0	0.0	0	0.0	2	0.4
Children with untreated tooth						
0	566	97.4	566	100	411	72.6
≧ 1	15	2.6	0	0.0	155	27.4
Children with a treated tooth						
0	581	92.3	566	100	526	92.9
≧1	0	0.0	0	0.0	40	7.1
Children’s caries status						
Caries-free	566	97.4	566	100	393	69.4
Caries	15	2.6	0	0.0	173	30.6
Mother with untreated caries						
No	–	–	–	–	406	71.7
Yes	–	–	–	–	89	15.7
No answer	–	–	–	–	71	12.5
Mother with treated caries						
No	–	–	–	–	150	26.5
Yes	–	–	–	–	345	61.0
No answer	–	–	–	–	71	12.5
Mothers with caries						
Caries-free	–	–	–	–	95	16.8
Caries	–	–	–	–	400	70.7
No answer	–	–	–	–	71	12.5
Total	581	100.0	566	100.0	566	100.0

Caries-free: having only healthy teeth and no missing teeth due to caries

Caries: having untreated or treated carious teeth

Mothers with caries: having at least one treated or untreated carious tooth when the child was seen for their checkup for 3-year-olds

At baseline, the mean age of the children was 18.5 months, with 95.2% ranging between 18 and 19 months of age. Just over half were boys. Nearly half (45.6%) were first in birth order and 41.0% were an only child. After 2 years at the checkup for 3-year-olds, the mean age was 42.8 months. Just 20.1% were still an only child.

At baseline, mothers were primary daytime caregivers for 68.6% of the children and a grandparent was a primary daytime caregiver for 40.1%. After 2 years, the percentage of mothers who were still the primary daytime caregivers decreased to 24.3% while grandparents increased to 78.2%. Among the 566 mothers at the checkup for 3-year-olds, only 16.8% had never experienced dental caries. In our sample, 61.0% of mothers reported that they had at least one treated previously carious tooth and 15.7% reported that they had at least one untreated carious tooth.

### Evaluations at baseline and at follow-up after 2 years

Analysis of dental evaluations at baseline showed that the number of erupted teeth ranged from 4 to 20, with a mean of 15.7 and median of 16. Over 60% of the children had 16 teeth. After about 2 years, the number of erupted teeth was 17–20, with a mean of 19.8 and median of 20. Over 94% of the children had 20 teeth. Among 18-month-old children, 52.5% children had 16 teeth and after 2 years almost all children had 20 teeth.

Two years later at the checkups for 3-year-olds, 155 children (27.4%) presented with one or more untreated carious teeth (maximum of 18 untreated teeth) and 40 (7.1%) with one or more treated previously carious teeth (maximum of 13 treated teeth) for a total of 173 children (30.6%) with one or more carious teeth (see Table 2). None of the children had a tooth missing due to caries. Among the 566 children, 85.0% had no plaque on the tooth surfaces of all teeth. This was a substantial improvement over baseline (44.9%) of plaque found on tooth surfaces of the maxillary central and lateral incisors. As compared with 47.2% at baseline who had a low level of plaque (i.e., at least one tooth with less than one third of the labial surface covered with plaque), at follow-up only 14.3% returned with a low level of plaque. None returned with a high level of plaque (i.e., at least one tooth covered in plaque on more than one third of the labial surface) as compared with 7.2% at baseline. Our sample of 3-year-olds at the study site had a higher prevalence of caries (30.6%) compared to those in Mie prefecture as a whole (18.6%).

**Table 2:** Status of teeth at baseline and follow-up (*n* = 566)

	Baseline		Follow-up	
	*n*	%	*n*	%
Number of children with one or more untreated teeth				
*d* = 0	566	100.0	411	72.6
*d* ≥ 1	–	–	155	27.4
Number of children with one or more treated teeth				
*f* = 0	566	100.0	526	92.9
*f* ≥ 1	–	–	40	7.1
Caries				
Never had caries	566	100.0	393	69.4
Had treated or untreated tooth	–	–	173	30.6
Plaque accumulation				
No plaque	254	44.9	481	85.0
Low level of plaque	267	47.2	81	14.3
High level of plaque	41	7.2	0	0.0
Not available	4	0.7	4	0.7

*d:* decayed primary teeth

*f:* filled primary teeth

Plaque accumulation at baseline on the surface of a child’s maxillary central and lateral incisors

Plaque accumulation at follow-up on the surface of any of a child’s teeth

Low level of plaque: covering less than one third of labial surface area of one or more teeth

High level of plaque: covering one third or more of a labial surface area of one or more teeth

### Associations between occurrence of caries, characteristics, and dental condition

To identify variables associated with the occurrence of caries after 2 years, we tested for correlations with child’s demographic characteristics, daytime caregivers’ characteristics, child’s dental health status, and mother’s dental health status (Table 3). We found only two variables that were significantly correlated with the occurrence of caries at follow-up: having low plaque (no children had high plaque at follow-up) and having a mother who had untreated caries at the time of follow-up.

**Table 3:** Characteristics, dental condition, caregivers status and caries at follow-up (*n* = 566)

	Caries-free		Caries		Total	*p* value
	*n*	%	*n*	%	n	
Gender						
Male	193	69.4	85	30.6	278	0.996
Female	200	69.4	88	30.6	288	
Position in birth order						
First	180	69.8	78	30.2	258	0.875
≥Second	213	69.2	95	30.8	308	
Variables at baseline						
Number of siblings						
0	160	69.0	72	31.0	232	0.840
≥ 1	233	69.8	101	30.2	334	
Mother is a primary daytime caregiver						
Yes	275	70.9	113	29.1	388	0.272
No	118	66.3	60	33.7	178	
Grandparent is a primary daytime caregiver						
Yes	154	67.8	73	32.2	227	0.501
No	239	70.5	100	29.5	339	
Plaque on a tooth surface						
None	173	68.1	81	31.9	254	0.606
Low/high	216	70.1	92	29.9	308	
Variables at follow-up						
Number of siblings						
0	76	66.7	38	33.3	114	0.473
≥1	317	70.1	135	29.9	452	
Mother is a primary daytime caregiver						
Yes	90	65.7	47	34.3	137	0.289
No	301	70.5	126	29.5	427	
Grandparent is a primary daytime caregiver						
Yes	309	70.1	132	29.9	441	0.469
No	82	66.7	41	33.3	123	
Plaque on a tooth surface						
None	351	73.0	130	27.0	481	< 0.001
Low	40	49.4	41	50.6	81	
Able to rinse of own mouth						
Yes	369	69.5	162	30.5	531	0.561
No	16	64.0	9	36.0	25	
Mother with untreated caries						
Yes	49	55.1	40	44.9	89	0.001
No	298	73.4	108	26.6	406	
Mother with treated caries						
Yes	240	69.6	105	30.4	345	0.693
No	107	71.3	43	28.7	150	
Mother with caries						
Caries	276	69.0	124	31.0	400	0.272
Caries-free	71	74.7	24	25.3	95	

Caries-free: only healthy teeth and no missing teeth due to caries

Caries: having one or more treated or untreated caries teeth

Plaque at baseline: on labial surface of one or more maxillary central and lateral incisors

Plaque at follow-up: on labial surface of one or more teeth

Low level of plaque: covering less than one third of labial surface area of one or more teeth

High level of plaque: covering one third or more of a labial surface area of one or more teeth

Mother with treated and untreated caries: when the child was seen for their checkup for 3-year-olds

Mother with caries: having a treated or untreated carious tooth when the child was seen for their checkup for 3-year-olds

*χ*^*2*^ test *p* value significance ≤ 0.05

### Health habits associated with the occurrence of caries

We also conducted tests to identify health habits associated with the occurrence of caries after 2 years (Table 4). At baseline, parents of 62.9% of children reported that a parent or the child brushed the child’s teeth every day, and of those, significantly more (72.7%) were caries-free at follow-up compared to those who did not do so every day (63.6%). At baseline, this was the only oral care habit significantly associated with not having caries. At follow-up, 59.2% of parents reported that they or their child brushed two or more times a day, while only 1.3% said not even once a day. At baseline, 89.7% of parents reported that they brushed their child’s teeth and at follow-up 93.1% reported doing so. At follow-up, 95.5% of children were able to rinse their mouth out with water on their own (see Table 3).

**Table 4:** Health habits associated with caries at follow-up (*n* = 556)

	Caries-free		Caries		Total	*p* value
	*n*	%	*n*	%	*n*	
Variables at baseline						
Brushing daily						
Every day	258	72.7	97	27.3	355	0.025
Not every day	133	63.6	76	36.4	209	
Anyone brushing						
Parent or child	384	69.9	165	30.1	549	0.463
Nobody	8	57.1	6	42.9	14	
Parent brushing						
Parent	351	69.5	154	30.5	505	0.853
Child only or nobody	41	70.7	17	29.3	58	
Visiting a dental clinic for checkup						
Regular	14	73.7	5	26.3	19	0.688
Irregular or no checkup	376	69.4	166	30.6	542	
Received fluoride application						
Yes	29	61.7	18	38.3	47	0.214
No	357	70.4	150	29.6	507	
Breastfed overnight						
Yes	50	58.8	35	41.2	85	0.018
No	341	71.6	135	28.4	476	
Bottle fed overnight						
Yes	11	57.9	8	42.1	19	0.282
No	369	69.5	162	30.5	531	
Snacking times						
As desired by child	132	62.0	81	38.0	213	0.002
Scheduled regularly	255	74.1	89	25.9	344	
Breastfed overnight and nobody brushing						
Breastfed and nobody	389	70.1	166	29.9	555	0.451
All other combinations	1	33.3	2	66.7	3	
Breastfed overnight and brushing regularly						
Breastfed and irregular	21	53.8	18	46.2	39	0.027
All other combinations	368	70.8	152	29.2	520	
Bottle fed overnight and brushing regularly						
Bottle fed and irregular	3	33.3	6	66.7	9	0.049
All other combinations	376	69.6	164	30.4	540	
Snacking and brushing						
No scheduled and irregular	49	52.7	44	47.3	93	< 0.001
All other combinations	337	72.8	126	27.2	463	
Variables at follow-up						
Brushing frequency						
≥ 2 times a day	229	69.8	99	30.2	328	0.926
< 2 times a day	146	70.2	62	29.8	208	
Anyone brushing						
Parent or child	382	70.1	163	29.9	545	1.000
Nobody	3	60.0	2	40.0	5	
Parent brushing						
Parent	360	70.3	152	29.7	512	0.557
Child only or nobody	25	65.8	13	34.2	38	
Visiting a dental clinic for checkup						
Regular	124	68.1	58	31.9	182	0.267
Irregular or no checkup	197	73.0	73	27.0	270	
Received fluoride application						
Yes	118	64.5	65	35.5	183	0.066
No	264	72.1	102	27.9	366	
Snacking times						
As desired by child	147	59.5	100	40.5	247	< 0.001
Scheduled regularly	224	77.5	65	22.5	289	
Snacking and brushing						
Not scheduled and less than twice daily	80	66.1	41	33.9	121	0.337
All other combinations	287	70.7	119	29.3	406	

Caries-free: only healthy teeth and no missing teeth due to caries

Caries: having one or more treated or untreated caries teeth

*χ*^*2*^ test *p* value significance ≤ 0.05

The percentage of children who had received a fluoride varnish application increased from 8.5% at baseline to 33.3% by follow-up. Those who received regular checkups at a dental clinic increased from 3.4% to 40.3%. These variables were not significantly associated with the occurrence of caries, although a child having had received a fluoride application by the time of the follow-up was nearly significant at *p* = 0.066.

Six of the nine dietary habit variables we measured were significantly associated with the development of caries between the baseline and follow-up. At baseline, 15.2% of children were breastfed overnight and 3.5% were bottle fed through the night. Bottle-fed children were given various liquids including powdered cow’s milk, liquid cow’s milk, and juice. The incidence of caries among children who breastfed overnight was significantly higher compared to those who did not breastfeed overnight (41.2% vs. 28.4%, respectively, *p* = 0.018). Additionally, at the baseline checkup for 18-month-olds, 38.4% of children had parents and daytime caregivers who did not regulate the frequency of snacking. At follow-up, the percentage increased to 46.1%. Among children seen at baseline, significantly fewer of those who had irregular snacking times were caries-free (62.0%) compared to those who had scheduled snack times (74.1%). The same was true among children seen at the checkup for 3-year-olds.

In bivariate tests of caries status versus interaction variables, “breastfed overnight and brushed irregularly” and “bottlefed overnight and brushed irregularly” were significantly associated with the development of caries. At baseline, 46.2% of children who were breastfed overnight and were not brushed every day and 66.7% of children who were bottle fed overnight and not brushed every day had at least one carious tooth. These percentages of caries children were significantly higher (*p* = 0.027 and 0.049, respectively) than “not breastfed overnight and/or brushed regularly” (29.2%) and “not bottlefed overnight and/or brushed regularly” (30.4%).

### Predictors of caries at 2-year follow-up

Our logistic regression analysis identified predictors of the development of caries at follow-up (Table 5). For this analysis, we excluded 12.5% of children whose mothers did not respond completely to the questionnaire. In our full model that included potential predictors identified in the literature along with interaction terms, the logistic regression analysis showed that the strongest significant predictors of having caries were the interaction of being bottle fed overnight and brushed irregularly at baseline (AOR 14.27), having plaque accumulation on one or more teeth at follow-up (AOR 2.41) and having a mother who had untreated caries at follow-up (AOR 1.84). The Nagelkerke *R*^2^ value for our model was 0.133.

**Table 5:** Logistic regression identifying predictors of having caries at follow-up (*n* = 463)

	Caries vs. caries-free (reference category)			
Predictor variables	OR (95% CI)	*p* value	AOR (95% CI)	*p* value
Male vs. female (reference)	0.85 (0.56–1.28)	0.434	0.71 (0.46–1.09)	0.117
Position in birth order^1^	1.03 (0.72–1.47)	0.875	0.89 (0.58–1.36)	0.588
Variables measured at baseline				
Parent(s) brushed child’s teeth^2^	0.93 (0.45–1.94)	0.846	0.70 (0.33–1.50)	0.361
Irregular visits to dental clinic for checkups	2.45 (0.54–11.21)	0.249	2.73 (0.58–12.94)	0.206
Breastfed overnight	1.77 (1.10–2.85)	0.019	1.85 (0.89–3.86)	0.100
Bottle fed overnight	1.66 (0.65–4.20)	0.287	0.36 (0.04–3.07)	0.347
Snacking unscheduled	1.76 (1.22–2.54)	0.003	1.15 (0.66–1.99)	0.621
Brushed irregularly	1.52 (1.05–2.19)	0.025	0.89 (0.46–1.73)	0.729
Breastfed overnight and brushed irregularly^3^	2.08 (1.08–4.00)	0.029	1.47 (0.48–4.53)	0.501
Bottle fed overnight and brushed irregularly^4^	4.59 (1.13–18.56)	0.033	14.27 (1.02–199.71)	0.048
Snacking unscheduled and brushed irregularly^5^	2.02 (1.15–3.54)	0.015	1.98 (0.81–4.86)	0.137
Variables measured at follow-up				
Unable to rinse own mouth	1.72 (0.67–4.37)	0.258	2.08 (0.79–5.46)	0.138
Plaque accumulation on one or more teeth^6^	3.30 (1.83–5.93)	< 0.001	2.41 (1.34–4.35)	0.003
Mother had untreated caries at follow-up	2.14 (1.28–3.59)	0.004	1.84 (1.09–3.12)	0.023

Caries-free: only healthy teeth and no missing teeth due to caries

Caries: having one or more treated or untreated caries teeth

^1^≥Second vs. 1st

^2^Child only or nobody vs. parent

^3^“Breastfed overnight” and “not brushing every day” vs. “not breastfed overnight” and/or “brushing every day”

^4^“Bottlefed overnight” and “not brushing every day” vs. “not bottlefed overnight” and/or “brushing every day”

^5^“Unscheduled snacking times” and “not brushing every day” vs. “snacking time scheduled regularly” and/or “brushing every day”

^6^Low level of plaque (covering less than one third of labial surface on one or more teeth) vs. none

To further investigate the variable of having a mother who had untreated caries at follow-up, we stratified children into three categories: (1) had a mother with untreated caries, (2) had a mother with treated caries, and (3) had a mother with caries. We then conducted bivariate analyses looking at associations between mothers’ caries status and several variables measure children’s dietary and oral health habits (Table 6). This analysis revealed that the only significant correlation was between the variables of having a mother with untreated caries and snacking times being unscheduled (i.e., having snacks whenever the child desired) such that 65.4% of mothers who did not have untreated caries also scheduled their child’s snacking regularly.

**Table 6:** Child’s health habits associated with mother’s dental health status

Variables at baseline	No		Yes		*p* value
	*n*	%	*n*	%	
Mother with untreated caries					
Breastfed overnight					
No	344	84.9	72	83.7	0.776
Yes	61	15.1	14	16.3	
Bottle fed overnight					
No	379	95.7	85	97.7	0.386
Yes	17	4.3	2	2.3	
Snacking times					
Scheduled regularly	263	65.4	42	48.3	0.003
As desired by child	139	34.6	45	51.7	
Daily brushing					
Every day	265	65.3	52	59.8	0.331
Not every day	141	34.7	35	40.2	
Mother with treated caries					
Breastfed overnight					
No	128	87.1	288	83.7	0.344
Yes	19	12.9	56	16.3	
Bottle fed overnight					
No	140	95.9	324	96.1	0.896
Yes	6	4.1	13	3.9	
Snacking times					
Scheduled regularly	95	63.8	210	61.8	0.675
As desired by child	54	36.2	130	38.2	
Daily brushing					
Every day	96	64.4	221	64.2	0.969
Not every day	53	35.6	123	35.8	
Mother with caries					
Breast fed overnight					
No	84	88.4	332	83.8	0.265
Yes	11	11.6	64	16.2	
Bottle fed overnight					
No	87	94.6	377	96.4	0.600
Yes	5	5.4	14	3.6	
Snacking times					
Scheduled regularly	65	69.1	240	60.8	0.131
As desired by child	29	30.9	155	39.2	
Daily brushing					
Every day	62	65.3	255	64.1	0.827
Not every day	33	34.7	143	35.9	

Mother with treated and untreated caries: when the child was seen for their checkup for 3-year-olds

Mother with caries: having a treated or untreated carious tooth when the child was seen for their checkup for 3-year-olds

*χ*^*2*^ test *p* value significance ≤ 0.05

## Discussion

The community we selected to study had a prevalence of caries among 18-month-old children at baseline that is reflective of the national prevalence in Japan (2.5% in 2009) [1]. However, 2 years later among 3-year-olds in the community, the prevalence of new caries (30.6%) was substantially higher than the prevalence of caries in 3-year-olds nationally over the same time period (23.0% in 2009; 21.5% in 2010; and 20.4% in 2011) [1]. Individual differences in ECC at 3 years of age were large; the numbers of untreated carious teeth per child ranged from 0 to18, and treated teeth per child ranged from 0 to 13. These data suggest that in this community, more children were at risk of developing ECC compared to their peers nationally.

In the present study, our multivariate analysis identified risk factors for ECC occurrence that were associated with children’s habits and mothers’ characteristics. In our initial model, four independent variables measuring dietary and brushing habits at 18 months of age did not load significantly by themselves: breast fed overnight, bottle fed overnight, snacking unscheduled, and brushed irregularly. Our subsequent model including interaction terms revealed that children who had been bottle fed overnight and brushed irregularly were 14.27 times more likely to have caries after 2 years than those who were not bottle fed overnight and were brushed or brushed themselves every day. This finding shows that a young child who is bottle fed overnight and not brushed/brushing themselves every day is at significantly increased risk of developing caries, most likely because of the extended contact time of sugary liquids on teeth combined with a lack of removal of resultant plaque that form on the surfaces of teeth leading to decalcification and ultimately dental caries. It is important to note that the significance of the interaction term suggests that this is a combined phenomenon.

Our study identified a second factor for ECC occurrence: the presence of plaque at age 3. Dental plaque has long been known as a risk factor for developing dental caries [31]. In our study, although the proportion of children with high levels of accumulated plaque decreased from 7.2% to 0% over 2 years, our analysis showed that children with even low levels of plaque were still 2.41 more likely to develop ECC compared to children who had no accumulated plaque. This finding adds a new dimension to findings reported in other studies that have shown that the longer plaque remains on the surface of primary teeth the higher the risk of developing ECC [10]. Bacteria in dental plaque decompose carbohydrates to produce acids that dissolve mineral components such as calcium present in teeth. The process of mineral decomposition along with the redeposition of the dissolved mineral (remineralization) occurs repeatedly, maintaining balance in the oral cavity. When this balance collapses, even with low levels of plaque, and decalcification becomes dominant and dental caries are formed.

From the complete formation of the primary teeth until the age of six when the eruption of permanent teeth begins, few changes occur in the dentition. Therefore, it is easy for parents and daytime caregivers to check and clean a child’s teeth. Our findings support the recommendation that primary caregivers should start the protective habit of brushing a child’s teeth as the first teeth erupt to remove the buildup of even low levels of plaque to prevent the development of dental caries [13]. Thus, parents should be advised to start brushing their child’s teeth twice daily after breakfast and dinner according to the eruption pattern and examine the child’s oral cavity on a daily basis. At checkups, the presence or absence of low levels of plaque can be used by health professionals to evaluate parents’ and daytime caregivers’ tooth brushing frequency and technique.

The third ECC risk factor we identified was a child having a mother with untreated caries. Children of mothers with untreated caries were 1.84 more likely to develop caries compared to children whose mothers were either caries-free or had treated caries. From our analysis, we believe that children whose mothers had untreated caries were more likely to have caries due to acquired reasons rather than because of an inherited trait. It is likely that a child’s oral health status was associated with their parents’ own oral health habits and understandings of the importance of developing effective oral hygiene skills with their children [32]. Child-rearing behaviors have been shown to be associated more strongly with ECC than mothers’ history of dental caries [33], and studies have shown that caregivers who visited a dentist primarily for checkups had children who did the same [34]. Few studies have identified an association between mothers’ untreated caries and ECC in their children [35]. Our study indicates that factors that cause a mother to develop caries (e.g., her diet, her dental self-care patterns, and barriers she faces to obtaining regular preventative dental care) are likely to also affect her child.

Fortunately, in Japan health checkups for children aged 18 months and 3 years are conducted in all municipalities according to the Maternal and Child Health Law [30]. Thus, public health nurses and health professionals can examine almost all children residing in Japan and their parents at least twice within 2 years. It is important for health professionals to be aware of protective and risk factors our study has identified so that they can provide parents and other caregivers with appropriate and meaningful oral health counseling. In terms of recommendations for more effective screening, the item “presence of dental plaque” is included in the oral examination and “use of a bottle” is a question item in the questionnaire for parents of 18-month-olds. However, there are no items to record a child being bottle fed overnight and not brushed regularly. Also, current screening does not identify mothers’ dental health status, particularly the presence of caries. To achieve higher levels of prevention and early detection of ECC, we recommend that these items be added to oral health screening in checkups for 18-month-olds and 3-year-olds.

This study has several limitations. First, the overall predictive success of the final model was somewhat low. Thus, there are likely to be other risk factors that our analysis could not identify. Second, our measures of dietary habits were somewhat general. We focused on the frequency of breastfeeding, bottle feeding, and snacking rather than on measuring specific dietary contents. Although intake of high-sugar snacks and/or beverages is well-established as a risk factor for ECC [10–12], we did not investigate the specific types of food and beverages or times of eating and drinking. Children in our study most certainly consumed various types of foods and snacks and various types of beverages in a bottle overnight (e.g., powdered milk, cow’s milk, juice) Thus, further longitudinal studies are needed to examine the interactions between specific foods and beverages and dental caries to identify specific dietary predictors of the occurrence of ECC among young children in Japan.

## Conclusions

To prevent ECC, greater public health efforts should be made in Japan to encourage parents to eliminate bottle feeding overnight and to promote brushing twice daily after breakfast and dinner as their children’s teeth begin to erupt. Greater efforts should be made to teach parents and daytime caregivers how to brush effectively to eliminate the buildup of even low levels of plaque. Health professionals should focus on these factors in health promotion, dental care counseling, and clinical care. Health professionals should also pay close attention to mothers’ oral health status to identify those with untreated caries. Mothers with caries should receive prompt dental treatment and also be assisted in developing better dietary and oral health habits that will benefit themselves and their children. Our recommendations can be used for screening items for being bottle fed overnight and not brushed regularly, and identifying caries in mothers can be used for health education and counseling for parents and daytime caregivers. Thus, public health policy and programs should focus more on family oral health rather than just child oral health.

## Abbreviations

CI: Confidence interval
ECC: Early childhood caries
OR: Odds ratio
